# Revisiting the functional annotation of TriTryp using sequence similarity tools

**DOI:** 10.1016/j.heliyon.2024.e39243

**Published:** 2024-10-11

**Authors:** Poorya Mirzavand Borujeni, Reza Salavati

**Affiliations:** a Institute of Parasitology, McGill University, Canada; b Department of Biochemistry, McGill University, Canada

**Keywords:** Functional annotation, Sequence similarity, Pseudogenes, Trypanosomatids

## Abstract

Trypanosomatids are the causative agents of deadly diseases in humans and livestock. Given the high phylogenetic distance of trypanosomatids from model organisms, these organisms have ample unannotated genes. Manual functional annotation is time-consuming, highlighting the importance of automated functional annotation tools. The development of automated functional tools is a hot research topic, and multiple tools have been developed for the task. PANNZER2 is an automated functional annotation tool that merely relies on the sequence similarity of the query to the annotated proteins. We tried PANNZER2 on *Trypanosoma brucei*, the most studied organism among trypanosomatids, to see if it could improve our knowledge of the functions of the genes.

Even with the availability of automated annotation tools like InterPro2GO in databases such as TriTrypDB, PANNZER2 has made surprisingly confident predictions for some hypothetical proteins in *T. brucei*. In this study, we identify gaps in such annotations because of not employing pairwise sequence alignment tools in TriTrypDB's automated annotation process. Our findings demonstrate that even the use of stringent cutoffs can successfully annotate a significant number of proteins. Additionally, we discovered that adjusting the open reading frames in certain genes leads to sequences with increased sequence signature coverage—characterized by the length covered by at least one sequence signature—compared to the original sequences. This enhanced sequence signature coverage suggests these genomic fragments could be pseudogenes. To facilitate further exploration, we developed a script to help identify potential pseudogenes within an organism's genome, offering researchers a new tool for genomic analysis and understanding. We extended all our analysis to *Trypanosoma cruzi* and *Leishmania major* to assess the impact of this approach across different species.

Our study demonstrates that by utilizing pairwise sequence similarity alignment, even with stringent cutoffs, we can attribute 2986, 3953, and 3798 new GO terms to the genomes of *T. brucei*, *T. cruzi*, and *L. major*. Additionally, we found that 210, 239, and 29 genes exhibit increased sequence signature coverage following frame correction, suggesting the presence of pseudogenes.

## 1 Introduction

Trypanosomatids are a group of unicellular eukaryotic parasites responsible for causing severe diseases in humans and animals [1]. Among these diseases, those of utmost clinical significance in humans include African sleeping sickness, caused by *Trypanosoma brucei gambiense* and *Trypanosoma brucei rhodesiense*, Chagas disease, caused by *Trypanosoma cruzi*, and various forms of Leishmaniasis caused by different *Leishmania* species [1]. Collectively known as TriTryps, these diseases can all lead to death if left untreated [1]. Each disease presents unique diagnosis, treatment, and control challenges, emphasizing the need for continued research and intervention strategies.

Effectively targeting a pathogen with drugs necessitates precise genomic annotation, beginning with the identification of gene locations and Open Reading Frames (ORFs) [2]. In this process, pseudogenes are also identified. Pseudogenes are non-protein-coding segments of DNA that resemble functional proteins. They are identified based on their sequence similarity to functional genes [3,4].

Following genomic annotation, the function of genes is predicted through a process known as functional annotation [2]. It is crucial to document these functions using a standardized language that the scientific community can universally understand. To meet this need, the Gene Ontology Consortium was established, providing a unified framework for representing gene functions [5]. Gene Ontology has three categories: Biological Process, Molecular Function, and Cellular Component. In the functional annotation of genes, the goal is to identify and report the most informative terms within each category for the genes of interest. For further insights into various methods of functional annotation, readers are encouraged to explore the 'Guide to GO Evidence Codes' available on the Gene Ontology Consortium's website [6].

Beyond augmenting our comprehension of a specific gene, the functional annotation of a gene significantly aids in annotating other genes within the same organism. More precisely, the physical interactions between an uncharacterized protein and proteins that have already been annotated, or the resemblance of its expression patterns to those of an annotated protein, may indicate shared biological roles [6]. Consequently, functional annotation sheds light on the roles of individual proteins and aids in the annotation of interacting genes or those with similar expression patterns, a concept known as guilt-by-association [7].

The experimental functional annotation of genes yields information of unparalleled quality, albeit at the cost of significant time and financial resources. Consequently, computational approaches to functional annotation have become critically important. Computational tools for functional annotation predominantly leverage the sequence or structural similarities between the query protein and those that have already been annotated [6].

While manual curation of functional annotations can enhance their accuracy, the ever-growing volume of biological data makes it impractical to curate all available data manually. For instance, at the time of writing this manuscript, only 572 thousand proteins in UniProt have undergone manual curation and are included in the Swiss-Prot database [8]. In contrast, over 240 million proteins remain uncurated and are included in TrEMBL, the non-expert curated section of UniProt [8]. As a result, automated annotation tools that bypass the need for manual curation, referred to as electronic annotation tools by the Gene Ontology Consortium [6], are particularly valuable.

According to the Gene Ontology consortium website, InterPro2GO is the most comprehensive and widely used method for electronic functional annotation [6]. InterPro2GO depends on InterPro, which houses a collection of sequence signature databases [9]. Some member databases, such as PRINTS, store sequence signatures of motifs [10]. Others, like Pfam, primarily store sequence signatures of domains [11], while databases like PIRSF focus on signatures of full-length proteins [12]. Each sequence signature is derived from multiple curated instances. InterPro analyzes the query sequences to identify patterns and associates each sequence signature with GO terms using the InterPro2GO table, an expertly curated resource.

The development of automated tools for functional annotation is an active area of research. To evaluate these tools, the Critical Assessment of Functional Annotation (CAFA) was established as a third-party initiative to benchmark and assess the performance of electronic annotation tools [13]. Five rounds of CAFA have been held so far. Yet, the published assessments are available only for the first three rounds [[13], [14], [15]].

In this study, we tried PANNZER2 [16], a leading contender in CAFA3 to see if it can improve the functional annotation of genes in *T. brucei*. PANNZER2 finds homologous sequences to the query, taking advantage of SANSparallel [17]. The hits with at least 40% sequence identity, whose alignment covers at least 60% of the query and subject, would pass the prefiltering step. Finally, the GO terms of the sequence neighborhood are attributed to the query using the ARGOT scoring function [16].

PANNZER2 relies on sequence similarity for its annotation process. Given that automated sequence-based functional annotation tools like InterPro2GO are integrated into TriTrypDB—a specialized database for trypanosomatids' data [18]—we did not anticipate making new GO term predictions. This study evaluated PANNZER2's predictions on *T. brucei*, the most extensively researched species among trypanosomatids. We conducted a manual review to pinpoint any deficiencies in TriTrypDB that might have led to missing annotations, to determine whether adding components to automated annotation pipelines could enhance annotation performance.

During our evaluation, we identified a genomic region that may have been incorrectly classified as a functional gene, as longer domains could be predicted for the same gene after adjusting its ORF. To address this issue, we developed a new script to predict potential pseudogenes in a genome by assessing the extent of a protein's coverage by predicted sequence signatures. We then applied our methods to *Leishmania major* and *T. cruzi*, two other clinically important trypanosomatids, to identify the affected genes.

## 2 Materials and Methods

### 2.1 Studied genomes

All the analysis has been performed on *T. brucei brucei TREU927*, *T. cruzi CL Brener Esmeraldo-like*, and *L. major* *strain Friedlin*, as these strains serve as reference organisms in TriTrypDB. Throughout the study, we utilized version 65 of TriTrypDB. The functional annotation of genomes downloaded from TriTrypDB was accessed via the GAF files.

For UniProt genome evaluation, the genome of *Trypanosoma brucei brucei (strain 927/4 GUTat10.1)*, proteome ID UP000008524, taxonomy ID 185431”, Genome ID and assembly GCA_000002445.1 was downloaded from UniProt. Functional annotation of the same genome was accessed by querying the taxonomy ID in UniProt and getting the table of all GO terms (accessed on 1 Sept 2024).

The ID mapping between UniProt and TriTrypDB IDs was performed using MMseqs2 in its easy-rbh mode on the proteomes downloaded from UniProt and TriTrypDB [19]. We only considered the hits with 100% sequence identity.

### 2.2 GO term manipulations

We used GOATOOLS for Gene Ontology term manipulations [20]. For all lists of GO terms, deprecated terms were updated to their current equivalents. The obsolete GO terms were removed from the dataset if no direct replacements were available.

The GO graph organizes terms hierarchically, where assigning a GO term to a gene also attributes all ancestor terms to that gene. We required a comprehensive and non-redundant list of GO terms in different steps. The extensive list of GO terms refers to a list containing attributed GO terms and all their ancestors, which was made using GoSubDag from GOATools. A non-redundant list of GO terms refers to a list that does not contain a GO term if one of its descendants is already on the list. To compile a list of non-redundant GO terms, we initially organized the GO terms associated with each gene by their depth within the hierarchy, arranging them in descending order. Subsequently, we added each GO term from this sorted list to our collection of non-redundant GO terms while eliminating all its ancestor terms. This process was repeated until the list lacked any remaining GO terms.

### 2.3 Functional annotation by PANNZER2

The proteome and GAF file for *T. brucei* *brucei* *TREU927* were obtained from TriTrypDB. The proteome was submitted to PANNZER2's web server for analysis on 5 March 2024 [16].

For finding new PANNZER2 predictions above TriTrypDB annotation, we looked for the attributed GO terms absent in the comprehensive list of TriTrypDB annotations. For evaluating PANNZER2 on the GO terms that are available in TriTrypDB but not UniProt, we looked at the GO terms that are available in comprehensive list of GO terms of TriTrypDB annotation but absent in the comprehensive list of GO terms of UniProt annotation. We calculated what fraction of such GO terms can be retrieved, relying on the comprehensive GO terms of PANNZER2.

### 2.4 Annotating based on similarity to a highly similar paralog/ortholog

The proteomes of *T. brucei*, *T. cruzi*, and *L. major* were each self-aligned using MMseqs2 [19]. We filtered out alignments where more than 90% of the query and subject sequences were aligned and exhibited a sequence identity above 70%. If a subject gene possessed GO terms supported by experimental evidence codes (EXP, IDA, IPI, IMP, IGI, IEP, HTP, HDA, HMP, HGI, or HEP) that were absent in the query's comprehensive GO term list, these instances were identified as potential new GO term predictions for the query gene.

For orthology-based annotation, we compared the proteomes of *T. brucei*, *T. cruzi*, and *L. major* against the proteomes of all organisms available in TriTrypDB. This was achieved by executing MMseqs2 in its easy-rbh mode [19]. We selected only those hits that met the criteria of having at least 80% alignment coverage for both the query and the target, along with a minimum sequence identity of 50%.

To quantify the specificity of each GO term, we calculated the information content using the Seco formula [21] by:

$$IC_{Seco}\;=\;1-\frac{\log\;\left(number\;of\;direct\;and\;indirect\;children\;of\;a\;GO\;term+1\right)}{\log\;\left(total\;number\;of\;GO\;terms\;in\;the\;GO\;namespace\right)}$$

In which, GO namespace could be a biological process, molecular function, or cellular component. Plots were visualized with matplotlib [22].

### 2.5 Finding the genes with an increase in sequence signature coverage after ORF correction

**Fig. 1:**
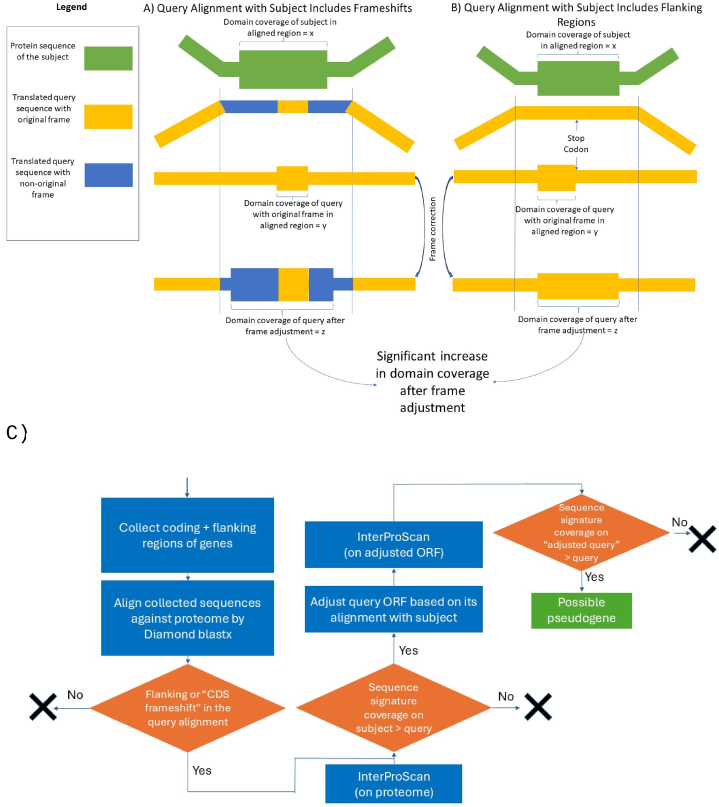
Schematic representation of: A) Genomic regions aligned with a subject protein, illustrating frameshift(s). B) Genomic regions aligned with a subject protein, extending into flanking regions (showing only the 3′ flanking region in this figure). In both A and B, aligned regions are depicted with parallel lines. The sequence signature coverage in the subject's aligned regions significantly exceeds that of the query. If the sequence signature coverage increases markedly after adjusting the query's frame based on its alignment with the subject, the query is marked as a potential pseudogene. C) Flowchart outlining the method employed, detailing the step-by-step process for analyzing sequence signature coverage and ORF adjustments.

Identifying genes that exhibit increased sequence signature coverage following ORF correction involves several key steps, summarized below. A detailed flowchart of our methodology is presented in Fig. 1C.

Step A : Selection of query-subject genes with possible query frameshift or truncation:

For each ORF, the coding sequence and 900 base pairs of flanking regions were gathered. These sequences were aligned with the organism's proteome using Diamond BLASTx in ultra-sensitive mode, utilizing the ‘-F 15 option to permit frameshifts in the alignment with a recommended penalty value of 15. We selected only genes with at least 50% of their coding region covered in the alignment.

If a portion of the query aligns with a different frame from the predicted frame within the subject protein's coding region (as illustrated in Fig. 1A) or if the alignment extends into the flanking regions (as shown in Fig. 1B), the sequence was advanced to the next step.

Step B : Sequence signature identification on the original sequence:

Using InterProScan [9] Version 95, we identified sequence signatures across the proteome of each organism by leveraging member databases such as Gene3D, Pfam, SUPERFAMILY, SMART, PRINTS, ProSiteProfiles, CDD, ProSitePatterns, PIRSF, Hamap, and SFLD.

Step C: Selection of query-subject pairs with greater sequence signature coverage on the subject:

For the aligned regions of both the query and subject, the sequence signature coverage—the length covered by at least one sequence signature—was calculated. We considered only those pairs where the sequence signature coverage on the subject was at least 20% and 20 units higher than that of query (with x indicating subject coverage and y indicating query coverage in Fig. 1). The query's ORF was adjusted based on its alignment with the subject in such cases.

Step D Sequence signature identification in adjusted ORF:

The adjusted ORF was analyzed for sequence signatures using the same approach as in Step A. If the sequence signature coverage of the adjusted ORF (denoted as z in Fig. 1) was significantly greater than the original ORF, the query was classified as a potential pseudogene. We specifically selected genes where the sequence signature coverage of the corrected ORF was at least 20 amino acids and 20% higher than in the original ORF of the query.

### 2.6 Pseudogene prediction with Pseudofinder

Pseudofinder [4] was executed on the proteomes of *T. brucei*, *T. cruzi*, and *L. major* using UniRef100 as the reference database. This tool requires the genome in GenBank format, which was created using the GFF file from TriTrypDB along with the genome and proteome data. Pseudofinder was executed with the --diamond option for increased speed. We only selected the predicted pseudogenes that overlapped with previously annotated genes.

### 2.7 Selection analysis

For the selection analysis of *T. brucei* and *T. cruzi* genes, we included all species from the *Trypanosoma* genus. At the same time, for *L. major*, we utilized all species from the *Leishmania* genus available in TriTrypDB. The complete list of selected organisms is provided in Supplementary Table 1. For *T. cruzi* *Sylvio X10/1*, two genomic annotations for the same genome are available. We selected the 2012 version because a greater number of its genes have orthologs in other species (verified by contacting the TriTrypDB team).

Although the adoption of syntenic orthologs for selection studies is generally recommended [23], we encountered instances suggesting that predicted syntenic orthologs may not be the optimal choice for our specific case. For instance, in TriTrypDB, the syntenic ortholog of Tb927.8.6960 in *T.* *brucei* *EATRO1125* is listed as Tb1125.Tb04.24M18.150, despite a notable difference in their lengths. In contrast, Tb1125.8.6960 in *T. brucei* *EATRO1125*, which matches Tb927.8.6960 in length and shows similarity in ID, appears to be a more accurate peer. To address such discrepancies, we found the Reciprocal Best Hits (RBHs) of proteins of the query organism in other species of the same genus using easy-rbh mode of MMseqs2 [19]. The list of all used organisms is available in Supplementary Table 1.

For genes with increased sequence signature coverage after frame correction, we aligned the subject-corrected sequences of the query genes, the subject itself, and the subject's RBHs using Muscle5 [24]. The coding sequences of the same proteins were codon aligned according to their protein alignment using pal2nal [25]. The maximum-likelihood phylogenetic tree of the genes was generated using the GTR + Γ4 model in PhyML [26], similar to what was explained in the original study of RELAX model [27]. RELAX model from HyPhy was used on the phylogenetic tree and codon-aligned sequences to investigate the relaxation of the selection [27].

## 3 Results and discussion

Our results reveal that 42,547 Gene Ontology (GO) terms are present in the comprehensive list of GOs provided by TriTrypDB that are absent from the UniProt comprehensive GO term list. Since PANNZER2 relies on the UniProt database [16], it does not have access to these specific GO terms, which presents an opportunity for further evaluation. Of the 42,547 mentioned GO terms, only 4,028 could be retrieved by PANNZER2 when predictions with a confidence score above 0.5 were considered. This discrepancy can be attributed to the relatively high phylogenetic distance of *T. brucei* from model organisms. Additionally, PANNZER2 uses SANSparallel for its analyses, which, although faster, is less sensitive than BLASTP for sequences that have diverged significantly [17].

PANNZER2 successfully predicted 400 new GO terms for 306 proteins in *T. brucei*, with each prediction having a confidence score above 0.8. Among these, “GO:0071076, RNA 3′ uridylation”, a biological process term, was attributed 19 times, making it the most frequently attributed GO term. According to QuickGO [28], “GO:0071076” is defined as “The enzymatic addition of a sequence of uridylyl residues at the 3′ end of an RNA molecule.” Of the 19 genes receiving this GO term, 13 were also annotated with “GO:0050265, RNA uridylyltransferase activity,” a molecular function term. Despite the lack of a direct link between “GO:0050265” and “GO:0071076” in the GO graph, QuickGO's website indicates that these two GO terms co-occur 96.59% of the time. Based on this observation, we conclude that all genes assigned “GO:0071076” can also be attributed with “GO:0050265,” and vice versa.

Among the six remaining genes not annotated with “GO:0050265,” three—KPAP1, KRET2, and TUT3—are known to have uridylyltransferase activity [29,30], emphasizing the importance of associating the appropriate GO terms with each studied function. Two of the proteins are not recognized as uridylyltransferases but are paralogs of MEAT1 and TUT4, which are known uridylyltransferases [29,30]. Notably, the sequence identities for these paralogs exceed 90%. This observation prompted us to hypothesize that TriTrypDB might lack an automated system for transferring annotations between a gene and its closely related paralogs. The TriTrypDB support team confirmed that there is no automated annotation transfer mechanism based on paralogy or orthology. We addressed this issue in a step that will be detailed later. It is important to note that “GO:0050265” could be potentially attributed to all five genes discussed in this paragraph. The remaining protein, Tb927.9.8780, has predicted domains almost exclusively indicating poly(A) polymerase activity. Consequently, we are uncertain whether Tb927.9.8780 possesses uridylyltransferase activity. A list of all genes predicted with “GO:0071076” is available in Supplementary Table 2.

Among the predictions, eight genes were identified with the GO term “GO:0042783, evasion of host immune response,” seven of which included Variant Surface Glycoprotein (VSG) in their description. The remaining protein is predicted to contain the SSF58087 domain, described as the “Variant surface glycoprotein (N-terminal domain).” VSGs are expressed on the surface of *T. brucei* and play a crucial role in evading the host immune system. Nearly 1000 pseudogenes have been identified on the main contigs of the *T. brucei* genome [31]. However, upon querying “GO:0042783” in TriTrypDB, we discovered that only 190 genes were annotated with this GO term, most of which were described as VSGs. In contrast, 419 functional proteins in *T. brucei* are labeled as VSGs. Consequently, we identified 251 functional proteins described as VSGs that lack the “GO:0042783” annotation. The list of these proteins is available in Supplementary Table 3.

### 3.1 Functional annotation based on pairwise sequence similarity

Considering the absence of an automated mechanism for transferring functional annotations from a gene to its paralogs, we investigated the potential extent of genes that could benefit from such annotations. Recognizing that other organisms within TriTrypDB face similar limitations, we expanded our analysis to include the proteomes of *T. cruzi* and *L. major*, alongside *T. brucei*. We conducted a self-alignment of the proteome for each organism to identify matches with genes carrying experimental annotations. Initially, our analysis did not impose any constraints on alignment coverage for the query or target sequences.

**Fig. 2:**
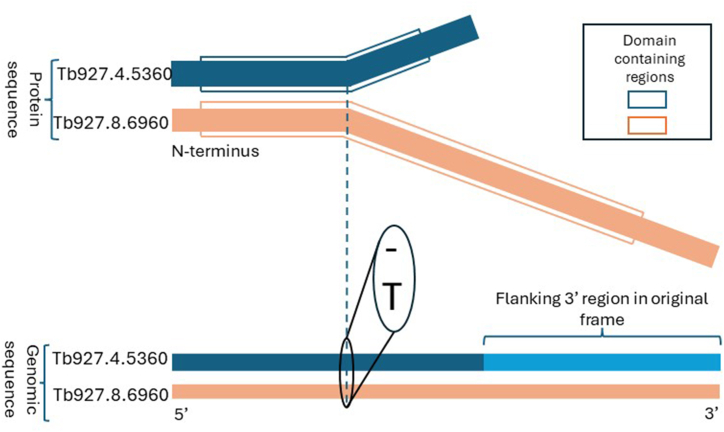
The schematic of protein and genomic sequence alignment between Tb927.4.5360 and Tb927.8.6960. Aligned regions of the protein sequences are shown with parallel boxes.

During our inspection, we came across Tb927.8.6960 (385 amino acids long), which aligns with Tb927.4.5360 (176 amino acids long). Tb927.4.5360 is annotated with “GO:0035082, axoneme assembly”, while Tb927.8.6960 does not have this GO term. The first 98 amino acids of the proteins align with each other with 95% sequence identity, and they differ significantly within the rest of their protein sequences. However, their genomic sequence alignment shows a high sequence identity throughout Tb927.8.6960 and Tb927.4.5360, plus the 3’ flanking regions. The genomic sequence alignment also shows a single nucleotide insertion/deletion at the position corresponding to the 99th amino acid, rendering the rest of the protein sequences dissimilar. The schematic of the alignments is shown in Fig. 2. Based on the alignments, Tb927.8.6960 or Tb927.4.5360 exhibits a frameshift, suggesting that one may be a pseudogene or contains a sequencing error. While acknowledging the potential for a sequencing error, we will refer to such cases as pseudogenes for simplicity in the upcoming discussions.

TriTrypDB shows that both proteins are predicted to have “SSF51905, FAD/NAD(P)-binding domain superfamily” domain. InterPro website shows that the sequence signature of the same domain has a length of 306. The mentioned domain stretches between positions 9 to 352 of Tb927.8.6960 and between positions 9 to 144 of Tb927.4.5360. Given the higher sequence signature coverage on Tb927.8.6960, we speculate that Tb927.8.6960 is functional and Tb927.4.5360 is a pseudogene. It is worth noting that even though both are classified as functional proteins in TriTrypDB, the transcript ID for Tb927.4.5360 in the same database is “Tb927.4.5360:pseudogenic_transcript”, supporting our hypothesis.

Unexpectedly, Tb927.4.5360 has experimental functional annotation. Yet, its function has been inferred in a study using RNAi for silencing target genes [32]. Given the genomic sequence similarity between Tb927.4.5360 and Tb927.8.6960, the RNAi silencing could have targeted both genes, meaning that the observed phenotype could have been due to the silencing of Tb927.8.6960.

From the alignment of Tb927.4.5360 with Tb927.8.6960, we drew two key conclusions. First, additional genes may exist where correcting the reading frame could enhance their sequence signature coverage; we will address this further in the following section. Second, when employing sequence similarity to infer functional annotations, ensuring that the aligned region encompasses a significant portion of the query and the subject is crucial.

To address the second concern, we aligned the proteome of each organism against itself as the next effort. We filtered the hits with at least 70% sequence identity and 90% alignment coverage threshold for both query and subject. Usually, 50% global sequence identity is high enough to infer functional similarity [33]. We selected a stringent threshold of 70% to minimize the false positive rate.

Furthermore, acknowledging the TriTrypDB team's clarification that there is no automated mechanism for annotation based on orthology relationships, we explored the potential for annotating genes through one-to-one orthology with all available organisms in TriTrypDB. We applied less stringent criteria for orthology-based annotation than those used for paralogy, selecting hits with a minimum of 50% sequence identity and 80% alignment coverage. The rationale for this approach is that the orthology relationship provides additional evidence, allowing us to be less stringent while still supporting the accuracy of the annotations.

**Fig. 3:**
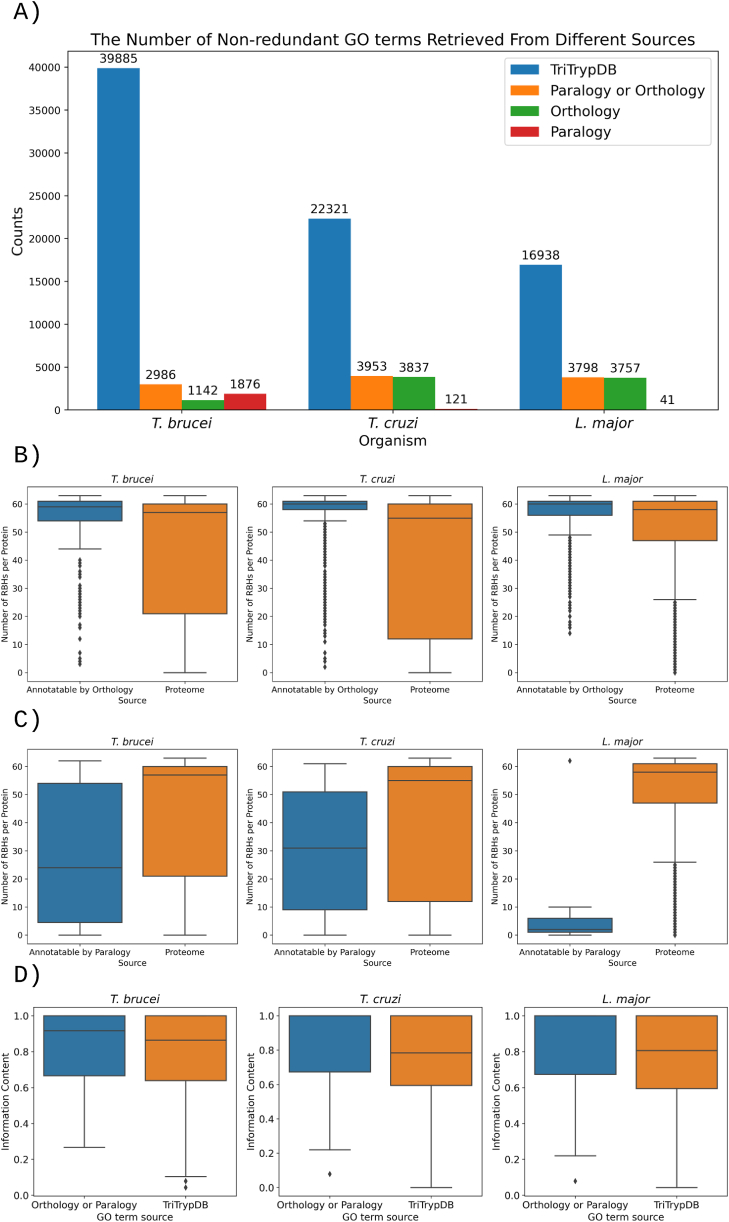
A) The number GO terms annotated based on orthology, paralogy, and the combined predictions of both. This also includes the count of GO terms available in TriTrypDB. B) The number of RBHs identified in TriTrypDB for genes annotated through orthology. C) The number of RBHs identified in TriTrypDB for genes annotated through paralogy. D) The information content of new GO annotations compared to the information content of existing annotations in TriTrypDB. Only non-redundant GO terms are considered for A and D.

Fig. 3A displays the number of annotations made using either paralogy or orthology. The minimal difference between the sum of annotations from each method and those obtained through their combination suggests that these annotations are largely non-overlapping. The figure also highlights the substantial impact that orthology/paralogy annotation can have on the number of GO terms in less-studied organisms, such as *T. cruzi* and *L. major*. Notably, for *L. major*, this approach can increase the number of annotated GO terms by up to 18.3%. The number of annotated genes and GO terms per namespace can be found in Supplementary Table 4. A comprehensive list of all annotations made through orthology and paralogy is available in Supplementary Table 5.

Fig. 3B illustrates that the number of Reciprocal Best Hits (RBHs) for proteins annotated by orthology is usually higher than the distribution for each protein across the entire proteome, suggesting these proteins are likely more conserved. In contrast, Fig. 3C reveals that the opposite is true for genes annotated through paralogy.

Information content measures, such as the Seco Information Content (IC_Seco_), can be used to assess the informativeness of GO terms. We used IC_Seco_ as it has demonstrated high performance in biological benchmarks [34]. IC_Seco_ ranges between 0 and 1, with higher values indicating richer information content in GO terms. Fig. 3D illustrates that the distribution of IC_Seco_ values for newly predicted GO terms is higher than those recorded in TriTrypDB, suggesting that annotations based on orthology/paralogy likely introduce more informative GO terms than those currently in TriTrypDB.

### 3.2 Finding putative pseudogenes among the predicted functional genes based on their sequence signature coverage

Initially, we aimed to identify frameshift mutations by analyzing the distribution of sequence signature signatures across different reading frames of the ORF currently predicted for each gene. This approach involved predicting sequence signatures in various frames; if a sequence appeared fragmented across different frames or if sequence signatures in different frames were predicted for an ORF, it suggested a frameshift mutation in the ORF, a concept akin to the strategy used by PseudoDomain [35]. However, we faced challenges when a frameshift occurred within a sequence signature, potentially making the fragmented sections of the sequence signature undetectable. Additionally, pinpointing the location of frameshifts using this method can be difficult. For instance, Tb927.4.5360, as previously discussed, has a frameshift before the 99th amino acid. Yet, the fragmented sequence signature on Tb927.4.5360 extends to the 144th amino acid, complicating the identification of the precise frameshift location.

To mitigate these issues, we explored the possibility of simulating frameshifts across different regions of the ORF to improve the identification of sequence signatures. However, this strategy introduces substantial computational demands owing to the vast array of potential mutated sequences. Furthermore, the probability of encountering multiple frameshifts within a single ORF significantly increases the computational burden, making this approach more challenging.

As previously mentioned, a functional gene, Tb927.8.6960, whose alignment with Tb927.4.5360 can indicate the presence of frameshifts, exists for Tb927.4.5360. Leveraging this insight, we developed a methodology to first adjust the ORF of the query based on its alignment with a homologous gene in the same genome and then check if sequence signature coverage improves because of the ORF adjustment. Our methodology has been detailed in Materials and Methods.

For comparison, we also used Pseudofinder [4] to predict potential pseudogenes in *T. brucei*, *T. cruzi*, and *L. major*. Pseudofinder was primarily developed for pseudogene identification in bacteria and archaea [4], and its annotation process relies on sequence alignments that could be incompatible with eukaryotic genomes due to the presence of introns. However, since most genes in trypanosomatids lack introns [36], their genomes in this respect are similar to bacterial and archaeal genomes. The regions marked as pseudogenes by our method always overlap with regions already annotated as genes and do not include pseudogenes located solely in intergenic regions. Therefore, we focused only on Pseudofinder predictions that overlap with pre-annotated genes.

### 3.3 Comparative analysis of pseudogene annotations

Based on the results of our method, six genes in *T. brucei* and one gene in *T. cruzi* despite having functional ORFs, exhibited increased sequence signature coverage after frame adjustment. This suggests that their ORFs were initially mispredicted. Consequently, these genes have been excluded from further analysis. Details about these genes and their corrected ORF locations can be found in Supplementary Table 6.

**Fig. 4:**
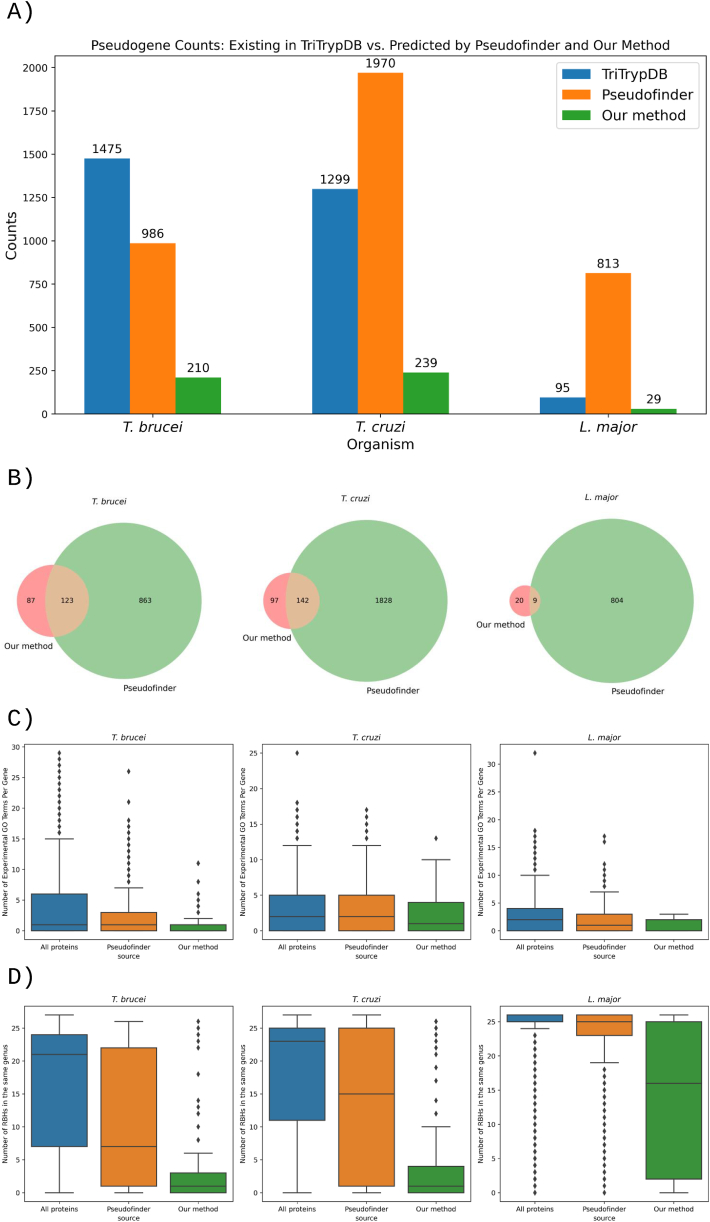
A) Comparison of the number of pseudogenes recorded in TriTrypDB with those predicted by Pseudofinder and our method beyond TriTrypDB entries. B) Venn diagram illustrating the overlap of pseudogenes identified by our method and Pseudofinder. C) Distribution of the number of experimental GO terms per gene for all proteins versus genes marked as putative pseudogenes by Pseudofinder or our method. D) Comparison of the number of RBHs found per gene for all proteins versus genes marked as putative pseudogenes by Pseudofinder or our method.

Fig. 4A compares the number of pseudogenes listed in TriTrypDB with those predicted by our method and by Pseudofinder beyond TriTrypDB annotations. Pseudofinder identifies a substantially higher number of genes as pseudogenes compared to our method. For *L.* *major*, which TriTrypDB has significantly fewer annotated pseudogenes compared to *T. brucei* and *T. cruzi*, our method also predicts fewer pseudogenes. However, Pseudofinder predicts several pseudogenes for *L. major* comparable to that for *T. brucei*.

Fig. 4B illustrates the overlap between pseudogenes predicted by our method and those predicted by Pseudofinder. Notably, the plot indicates that for all organisms, at least 40% of pseudogenes predicted by our method were not identified by Pseudofinder.

Although pseudogenes have occasionally been shown to gain new regulatory functions [37], they cannot encode functional proteins [38]. Consequently, they are typically not analyzed in experimental functional annotation projects. Fig. 4C shows that the distribution of experimental GO terms assigned to regions identified as pseudogenes by our method is lower than those identified by Pseudofinder. Furthermore, the distribution of experimental GO terms linked to pseudogenes identified by Pseudofinder is lower than the those assigned to the complete proteome of each organism.

Pseudogenes experience lower selective pressure, which allows them to mutate more rapidly. As a result, they are expected to have fewer orthologs compared to the entire proteome, which serves as a background. Fig. 4D demonstrates that the distribution of the number of RBHs identified for each gene is lower for the regions we have predicted as pseudogenes compared to those identified by Pseudofinder. Additionally, the RBH distribution for Pseudofinder's predictions is lower than that of the entire proteome. A direct comparison of Pseudofinder and our method, based on Fig. 4C and D, suggests that our predicted pseudogenes likely have a lower false positive rate than those identified by Pseudofinder, as their characteristics more closely resemble true pseudogenes.

### 3.4 In-depth examination of genes labeled as pseudogenes using our workflow

In *T. brucei*, the three most common protein descriptions observed were VSG, hypothetical protein, and retrotransposon hotspot protein, appearing 99, 26, and 8 times, respectively. An early study found that 86% of VSGs in *T. brucei* are pseudogenes [31]. Additionally, retrotransposon hotspot proteins are known to be prone to becoming pseudogenes [39].

In *T. cruzi*, the top three most common protein descriptions were hypothetical protein, trans-sialidase, and surface protease GP63. According to TriTrypDB annotations, trans-sialidase, and surface protease GP63 are frequently observed among pseudogene descriptions in *T. cruzi*, with 493 and 106 pseudogenes, respectively, described as such.

For *L.* *major*, 10 genes labeled as pseudogenes in our workflow were described as hypothetical proteins. We did not observe any discernible pattern in the remaining genes.

The distribution of pseudogenes in *T. brucei* appears to be highly concentrated on the “Tb927_11_bin_v5.1” contig, with 152 out of 210 predicted pseudogenes located there. This is a significant concentration, especially when compared to the next contig, “Tb927_11_v5.1,” which predicted to have only 12 pseudogenes. In contrast, for *T. cruzi* and *L. major*, no similar pattern of pseudogene distribution is observed.

In *T. brucei*, *T. cruzi*, and *L. major*, 58, 93, and 7 predicted pseudogenes, respectively, exhibit over 80% sequence identity with the subject sequences used for their ORF adjustments. Typically, once a gene becomes a pseudogene and is no longer under selective pressure, we anticipate it will rapidly diverge from its functional counterpart. Thus, the high sequence similarity increases the likelihood that these sequences might contain sequencing errors rather than being true pseudogenes. However, in *T. brucei*, for eight genomic fragments with high sequence similarity to their subjects, RBH with a sequence identity above 80% were identified in *T. brucei* *EATRO1125*, a closely related species to *T. brucei* *TREU927*. This finding suggests that these sequences are likely genuine and not the result of sequencing errors.

The RELAX model provides an intensification parameter, denoted as k, which quantifies the relaxation or intensification of selection. Values of k greater than 1 indicate intensified selection, while values less than 1 suggest relaxed selection, typically expected in pseudogenes. In *T. brucei*, for 64 of the selected genes, and in *T. cruzi*, for 25, the p-value of RELAX is significant (less than 0.05). In *L.* *major*, none of the genes show significant results. Among the significant findings, 42 genes in *T. brucei* and 18 genes in *T. cruzi* exhibit k values smaller than 1, indicating relaxed selection.

### 3.5 Current limitations and future improvements of our pseudogene annotation method

The reliability of sequence signatures identified by different tools is critical for our workflow. The same is true for identifying pseudogenes based on sequence similarity to functional genes [3,4]. As each sequence signature database is manually curated, we believe there is a lower chance of having spurious sequence signatures than cases where comprehensive un-curated genomic sequences are used as the reference. Still, spurious sequences and ORFs had been occasionally integrated into sequence signature databases [40].

Recognizing the possibility of partial false positives in sequence signature annotation tools is essential. For instance, the protein Tb927.4.5360, which we discussed previously, shows a frameshift before the 99th amino acid, yet the predicted sequence signature extends to the 144th amino acid. This suggests that the sequence signature prediction from the 99th to the 144th amino acid might be a partial false positive. Nonetheless, our study maintains a low false positive rate as the genes have undergone rigorous filtration criteria. Each gene paired with a homologous template to correct ORF, implies that the homolog could potentially compensate for the functions of any disrupted gene. Moreover, we concentrated on genes that demonstrated a significant increase in sequence signature coverage following ORF correction compared to their original frames. While false positives might appear in the corrected and original ORFs, their differences could negate one another. Our methodology, therefore, likely achieves a lower false positive rate than techniques that find pseudogenes by identifying genomic fragments with sequence signatures annotated across different frames.

For a pseudogene to be captured by our method, a homolog must be present, and the sequence signature coverage of the query must increase after adjustment of its ORF based on its alignment with the homologous sequence. Due to these requirements, our method cannot capture the pseudogenes that do not have a homologous sequence in the genome. Although, in theory a comprehensive database can be used to correct the ORF of query pseudogenes, as InterProScan must be run on every adjusted ORF, the computational cost for our method would be prohibitive. Furthermore, if no sequence signature can be predicted for the adjusted sequence, our method will miss the pseudogene.

Besides, as our tool relies on the adjustment of the query based on its alignments with a homologous gene, it will fail if the alignment of a query with the target is interrupted by noncoding sequences, for example, because of the existence of introns. This is not a major issue in trypanosomatids, as only a few genes possess introns [36]. However, in other eukaryotes, our pipelines can only identify processed pseudogenes, the genomic regions that are the result of processed mRNA retrotransposition [38].

Here, we used sequence signature coverage to uncover the pseudogenes among the list of functional proteins. The foldability of the region can also be used for pseudogene identification. AlphaFold shows its confidence in predicting the structure of a residue with pLDDT, a number between 0 and 100 where higher values show higher confidence [41]. Average pLDDT, or the number of residues with a pLDDT above a specific threshold can also be used for finding pseudogenes instead of sequence signature coverage. However, replacing the structure-derived numbers with sequence signature coverage requires prediction of the structure of adjusted ORFs which needs quite heavy computations.

## 4 Conclusion

In this study, leveraging a sequence-based functional annotation tool, we identified gaps in protein databases that, when addressed, could enhance the functional annotation of organisms. We discovered several hypothetical proteins with highly similar annotated counterparts, underscoring the potential of transferring annotations from one gene to its similar copies to label unannotated genes. Although these discrepancies are often readily correctable, they significantly impact many genes. This research is critical as many high-throughput studies rely on interactions between known and unknown proteins to infer function. Comprehensive and accurate gene annotations are essential for these studies, especially since researchers often lack the time to manually verify and update database entries.

Additionally, we found GO predictions supported by external experiments that have yet to be integrated into TriTrypDB, highlighting the need for continuous updates to the GO annotation of each organism to reflect newly discovered functionalities.

Moreover, we developed a script to identify genes where ORF correction significantly increases sequence signature predictions. We hypothesize that such ORFs might be pseudogenes, suggesting that some hypothetical genes in lesser-studied organisms may be pseudogenes read in incorrect frames, resulting in protein sequences that diverge from known patterns.

## CRediT authorship contribution statement

**Poorya Mirzavand Borujeni:** Writing – original draft, Methodology, Investigation, Formal analysis. **Reza Salavati:** Writing – review & editing, Supervision, Project administration, Funding acquisition.

## Data availability

The scripts used for the identification of pseudogenes are available at https://github.com/Pooryamb/PseudoGene.

## Declaration of generative AI and AI-assisted technologies in the writing process

While preparing this manuscript, the authors utilized GPT-4o to enhance readability and optimize certain script processes. Subsequently, the authors thoroughly reviewed and edited the content as necessary, taking full responsibility for the final published article.

## Declaration of competing interest

The authors declare the following financial interests/personal relationships which may be considered as potential competing interests: Reza Salavati acknowledges the financial support provided by the 10.13039/501100000024Canadian Institutes of Health Research under grant number 252733. If there are other authors, they declare that they have no known competing financial interests or personal relationships that could have appeared to influence the work reported in this paper.
